# Dr. Robert Leamon Bowles

**Published:** 1913-11-29

**Authors:** 


Dr. Robert Leamon Bowles, of London and Folkestone, has died at the age of seventy-nine. Educated at St. George's Hospital, he gained his F.R.C.P. in 1882, and became consulting physician to Folkestone Hospital. A publicist of wide range, he wrote the article on "Stertor" for Quain's Dictionary of Medicine, and also an article on "Sunburn" in the *Alpine Journal* in 1888, which is interesting in the light of his contributions on the influence of light upon the skin. He had been a Gloucestershire magistrate.

